# Ohio State Dental Society

**Published:** 1885-11

**Authors:** 


### EDITORIAL.

### Ohio State Dental Society—Reorganization.

The first annual meeting of this body was held on the 28th and the 30th inclusive. The principal work of this meeting, was the reorganization of the Society, the arranging and adopting of a Constitution and By-Laws The committee who had been appointed for this work, presented an excellent report, which, with but few changes, was unanimously adopted. Great care, however, was exercised in this work, the experiences derived in the old Society were valuable, as indicating what should, and should not be done. This was done, of course, by the eighteen incorporators, who one year ago secured articles of incorporation. All of the members who were present, were invited to participate in the discussion, and give suggestions in reference to the organization. After the adoption of the Constitution and By-Laws, the admission of members was in order. The application for membership was on blanks prepared for the purpose, and recommended by one or more of the active members. The admission to membership requires four-fifths of the votes cast, in order to elect. There were forty-three members elected and enrolled, which was considered as very encouraging indeed for the future of the Society, in as much as there had been many misgivings in regard to the success that might attend the organization; there seems to be in the minds of some of the Profession, incorrect views in reference to it, and in the minds of many others, a feeling of uncertainty as to the course that would be pursued. But for these two influences, the attendance undoubtedly would have been much larger, still, as it was, the attendance was about as large as it has been at the old Society for the past four years; there were in attendance in all, between sixty and seventy. In addition to the work just referred to, the subject of amendment to the Dental Law was fully discussed; the importance of change was conceded by all. A draft of the law or amendment was presented and freely considered. There was entire unanimity as to the features that should be embodied in the law, and very little difference of opinion as to the language in which they should be couched. A committee was appointed who should have this matter in charge and do whatever might be necessary to secure the passage of the enactment. In addition to the work already mentioned, several papers were read, most of which elicited marked interest, however, but little time was afforded for their discussion. Quite a number of papers had been prepared, for which time was not afforded for the reading; there was no time for clinics or a public exhibition of instruments and appliances; there was, however, quite a number of such things that were seen by, and explained to the members individually. Chillicothe proved to be an excellent place for the meeting. Quite as good, if not better accommodations were afforded for both meetings and the members as at former times, so good indeed were these that there was quite a general opinion in favor of returning at the next meeting; however, it was finally decided to hold the next meeting in Toledo, and it will be held on the last Tuesday of October, 1886, at which time there is a strong probability, amounting almost to certainty, that there will be a large and enthusiastic meeting; of this there is no doubt, if the Profession in the State, generally, becomes informed fully, as to the character of this meeting. The strong desire and expectation of all those who were present, is that the future of the Ohio State Dental Society will be a successful and brilliant one—so may it be.

